# Temperature Perception Regulates Seed Germination in *Solanum nigrum* via Phytohormone Signaling Pathways

**DOI:** 10.3390/ijms262311757

**Published:** 2025-12-04

**Authors:** Ziqing Ma, Lu Yang, Zhihong Feng, Longlong Li, Kaidie Wu, Yue Xiong, Hongjuan Huang, Shouhui Wei

**Affiliations:** State Key Laboratory for Biology of Plant Diseases and Insect Pests, Institute of Plant Protection, Chinese Academy of Agricultural Sciences, Beijing 100193, China

**Keywords:** *Solanum nigrum*, RNA-seq, germination response, temperature signal, regulatory pathway

## Abstract

Black nightshade (*Solanum nigrum* L.) is a highly invasive weed in agricultural systems, primarily dispersed by seeds. Its germination is regulated by temperature, which varies among populations. We investigated the germination responses to temperature in two *S. nigrum* populations (XJ1600 and XJ1633) and identified eight putative candidate genes: *GA20ox1*, *GA3ox1*, *GA2ox1*, *NCED9*, *CYP707A2*, *SPT*, *PIF1*, and *ABI5*. These genes are involved in the phytohormone signaling pathway. Under low-temperature conditions, *SPT* likely perceives cold signals, promoting germination by up-regulating *GA20ox1* and *CYP707A2* while suppressing *GA2ox1*, thus potentially increasing bioactive GAs and reducing ABA levels. Under high-temperature conditions, *PIFs* likely perceive the heat signal. Through regulation mediated by the known negative regulator *SOMNUS* (*SOM*), *NCED9* and *GA2ox1* were up-regulated, while *GA3ox1* was down-regulated, which could collectively modulate seed germination by altering ABA and GA levels. This study clarifies how temperature regulates *S. nigrum* seed germination through integrated hormonal and genetic mechanisms. This understanding directly supports the development of predictive models for weed emergence and informs the design of sustainable control strategies.

## 1. Introduction

*Solanum nigrum* (black nightshade) is an annual broadleaf weed that belongs to the *Solanaceae* family, is widely distributed across more than 60 countries, and is known to infest over 30 crop species [1,2,3]. As a problematic weed in Chinese cotton (*Gossypium hirsutum* L.) fields, *S. nigrum* competes with cotton for resources and poses a significant threat to crop quality. During harvest, its berries are easily crushed, staining the cotton fibers and reducing their market value. Furthermore, the weed’s high fecundity and persistent soil seed bank allow its seeds to remain viable and germinate over multiple seasons. While mechanical and chemical methods are the most widely adopted control strategies, their long-term efficacy is diminishing. The persistent use of the same herbicides, combined with the widespread adoption of drip irrigation under plastic mulch, has recently led to a rapid increase in *S. nigrum* infestations in cotton and maize (*Zea mays* L.) fields. This weed is transitioning from a common species to a dominant one, posing a significant threat to crop productivity. Consequently, effective management of *S. nigrum* is essential for ensuring high-quality agricultural output [1].

Seeds represent the beginning of the plant life cycle [4,5]. Seed germination is a critical, yet complex, physiological process, and in nature, only a small proportion of seeds succeed in germinating [6]. This low germination rate is frequently attributable to seed dormancy, a common adaptation wherein seeds must break dormancy to complete germination [7,8]. The germination process is influenced by multiple environmental factors, including moisture, temperature, light, and oxygen [9,10]. These factors mediate the interaction between seeds and their ecological environment, ultimately determining the ability of plant populations to disperse, occupy new areas, and sustain renewal through reproduction [11,12,13]. Among these, temperature is a critical determinant, as it directly modulates both the rate and speed of germination [14]. For instance, low temperatures can prevent internal enzymatic activities from reaching sufficient levels, thereby delaying the germination process [15,16]. Conversely, high temperatures may inactivate these essential enzymes and even induce secondary dormancy, effectively preventing germination [17]. Previous studies have shown that the optimal temperature for the germination of *S. nigrum* seeds from Xinjiang is 30 °C, and the temperature range for germination is between 15 and 35 °C [18]. This range is consistent with reports for other solanaceous weeds, such as *Solanum sarrachoides* and *S. elaeagnifolium*, which also exhibit optimal germination around 25–30 °C and significant inhibition at 10 °C [19,20]. Furthermore, our temperature selection aligns with the classic physiological framework of cardinal temperatures for seed germination [20]. Critically, our phenotypic screening confirmed 30 °C as the peak temperature for the germination index and GA/ABA ratio, 15 °C as sub-optimal, yet permissive, and 10/35 °C as inhibitory stresses. Thus, the 15/30 °C contrast was used for transcriptomic discovery, while 10/35 °C served to validate the expression and putative function of the candidate genes under abiotic stress.

Environmental factors regulate seed germination by modulating signal transduction, biosynthesis, and metabolic pathways, thereby inducing dynamic changes in the balance of endogenous hormones [8,21,22]. However, the precise regulatory mechanisms through which these factors control dormancy and germination remain to be fully elucidated [8,23]. Abscisic acid (ABA), a sesquiterpenoid phytohormone, plays pivotal roles in modulating seed dormancy and integrating environmental cues—particularly temperature signals—to mediate abiotic stress responses [24,25]. In contrast, gibberellic acid (GA), a diterpenoid phytohormone, promotes germination by enhancing enzymatic activity and stimulating embryonic growth [26]. When seeds perceive unfavorable temperatures, the hormonal balance shifts: ABA biosynthesis is up-regulated, while the conversion of metabolic intermediates to GA is suppressed. This interplay reinforces dormancy and effectively inhibits germination [27,28]. Previous studies indicated that the phytohormone content during the seed germination of *S. nigrum* showed a trend of increasing GA content and decreasing ABA content to promote the germination [29,30]. The influence of temperature on this hormonal balance is further evident at the molecular level. For instance, in *Arabidopsis* seeds, low temperature has been shown to up-regulate the expression of GA biosynthesis genes while down-regulating those involved in GA catabolism [31,32,33,34,35]. Consistent with this, the over-expression of *MAPK11* (*MITOGEN-ACTIVATED PROTEIN KINASE 11*) affected the ABA signaling pathway and ultimately enhanced the germination of tomato seeds by phosphorylating *SnRKs* at low temperatures, whereas *MAPK11* might affect *GA20ox* and *GA2ox* to regulate seed germination indirectly by promoting GA biosynthesis [36,37,38]. Collectively, these studies show that temperature influences seed germination by fine-tuning the metabolic and signaling pathways of ABA and GA.

However, the mechanism by which temperature perception regulates seed germination through phytohormone pathways in *S*. *nigrum* is poorly defined. To investigate this, we employed a two-step approach: first, by identifying candidate genes at the optimal temperature (15/30 °C), and then, by exploring their responses under stressful temperatures (10/35 °C) to characterize their potential role in temperature adaptation. This work thus connects temperature perception to phytohormone signaling in *S. nigrum*, providing molecular groundwork for future germination-based weed management strategies.

## 2. Results

### 2.1. Germination Difference

**Figure 1:**
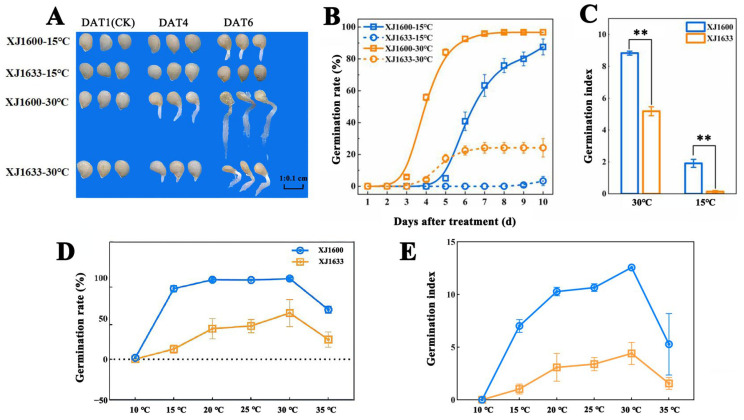
Germination of *Solanum nigrum* populations XJ1600 and XJ1633 at 15 °C and 30 °C. (**A**) Germination behavior of two *S. nigrum* populations; (**B**) germination percentage of two *S. nigrum* populations over time; (**C**) germination index of two *S. nigrum* populations; (**D**) seed germination rates of two *S. nigrum* populations (XJ1600 and XJ1633) between 10 °C and 35 °C; and (**E**) seed germination index of two *S. nigrum* populations (XJ1600 and XJ1633) between 10 °C and 35 °C. Vertical bars represent the standard error of the means; ** represents significant differences at the *p* ≤ 0.01 level.

The germination responses of two *S. nigrum* populations (XJ1600 and XJ1633) to distinct temperatures (15 °C and 30 °C) are presented in Figure 1. Germination was defined as the radicle emerging ≥1 mm from the seed coat. During imbibition under 30 °C, seeds of XJ1600 population started to germinate 3 days after treatment (DAT), and the daily germination rate peaked at 50% at 4 DAT and stabilized with a germination rate of about 95% at 7 DAT, while XJ1633 started to germinate at 4 DAT and stabilized with a germination rate of about 25% at 7 DAT (Figure 1B). During imbibition under 15 °C, the germination rate and speed of XJ1600 were significantly higher than those of XJ1633, and the germination rate increased continuously from 6 to 10 DAT, with the maximum rate reaching 87.5% at 10 DAT, while XJ1633 showed almost no germination at this temperature. The germination index serves as an indicator of both germination speed and uniformity in seeds, with higher values corresponding to faster and more consistent germination [39]. In this experiment, XJ1600 population germinated faster and were more uniform, while seeds of XJ1633 germinated more slowly (Figure 1C).

The germination rates of both XJ1600 and XJ1633 showed an initial increase, followed by a decrease, between 10 °C and 35 °C. At 10 °C, the germination rate was almost negligible for both *S. nigrum* seeds. Germination gradually increased at 15 °C and reached its peak at 30 °C (Figure 1D). Even with 60% germination at 35 °C, high-temperature stress responses were triggered, partially overlapping with those at the optimal 30 °C. The germination index followed a similar trend, also peaking at 30 °C (Figure 1E). Since 15 °C and 30 °C resulted in the lowest and highest germination indices, respectively, and showed the most significant difference in germination rates (with the GA/ABA ratio also peaking at 30 °C), the 15/30 °C temperature combination is ideal for studying the effects of temperature on germination.

### 2.2. Seed GA and ABA Content

**Figure 2:**
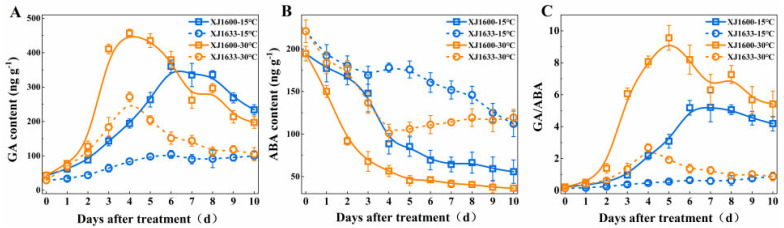
Content of internal phytohormones GA and ABA in *S. nigrum* during seed imbibition. (**A**) GA; (**B**) ABA; and (**C**) changes in the GA/ABA ratio. Vertical bars represent the standard error of the means.

Phytohormones in seeds, particularly GA and ABA, play pivotal roles in regulating germination. In our experiment, all samples for hormone measurement were harvested at the same time points across all experimental groups. The key time points for comparing XJ1600 and XJ1633 correspond to comparable developmental stages. During the initial germination phase, the GA content in both XJ1600 and XJ1633 seeds increased significantly, peaking at 4 DAT, with values of 456.42 ng/g and 271.12 ng/g, respectively, under 30 °C conditions, followed by a gradual decline. On the other hand, at 15 °C, the seed GA content of these populations peaked at 6 DAT (Figure 2A). The ABA content in seeds exhibited a gradual decline as imbibition time, with the highest level (194.52–220.77 ng/g) detected prior to the onset of imbibition (Figure 2B). During imbibition at 30 °C, XJ1600 seeds exhibited a lower initial ABA content, which decreased rapidly within the first two days and reached its observed minimum at the organ-wide level by 10 DAT. By comparison, XJ1633 seeds had a higher initial ABA content and a slower decrease, retaining a relatively higher ABA content at 10 DAT. The GA/ABA ratio in the XJ1600 population exceeded 1.0 at 2 DAT under 30 °C, surged to 6.0 by 3 DAT, and peaked at 9.5 at 5 DAT (Figure 2C). On the other hand, at the lower temperature of 15 °C, the GA/ABA ratio of XJ1633 seeds remained below 1.0 throughout the entire imbibition period. This study has a limitation: measuring hormone levels at 10 °C and 35 °C would have made the experimental design more robust.

### 2.3. Sequencing Quality and Reads Assembly

**Figure 3:**
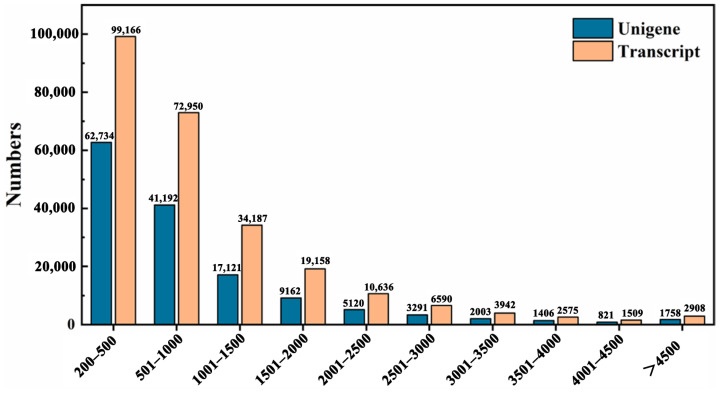
Distribution of unigene and transcript length for transcriptome sequencing of *S. nigrum* seeds.

To gain insights into the transcriptional responses and identify candidate genes underlying *S. nigrum* seed germination in response to temperature variations, we performed deep sequencing of cDNA libraries derived from 36 seed samples subjected to 15 °C and 30 °C treatments using the Illumina platform Software Version: 3.7.5. Following the removal of adapter sequences, low-quality reads, and ambiguous bases, a total of 245.86 GB high-quality clean reads were obtained from the cDNA libraries, with each sample yielding over 6.00 GB of clean data. All samples exhibited high-quality sequencing data, with Q20 and Q30 scores exceeding 97.33% and 92.43%, respectively. These results demonstrate both the sufficient quantity and reliability of the data, supporting their suitability for downstream analysis in this study. Clean reads were assembled in Trinity and yielded a total of 253,621 transcripts and 144,608 unigenes. The comparison rate for mapping analysis was 69.90–78.68%, which indicates a high quality of the assembly. All unigenes exceeded 200 bp in length, including 62,734 unigenes spanning 200–500 bp and 40,682 unigenes longer than 1000 bp (Figure 3).

### 2.4. Functional Annotations

**Figure 4:**
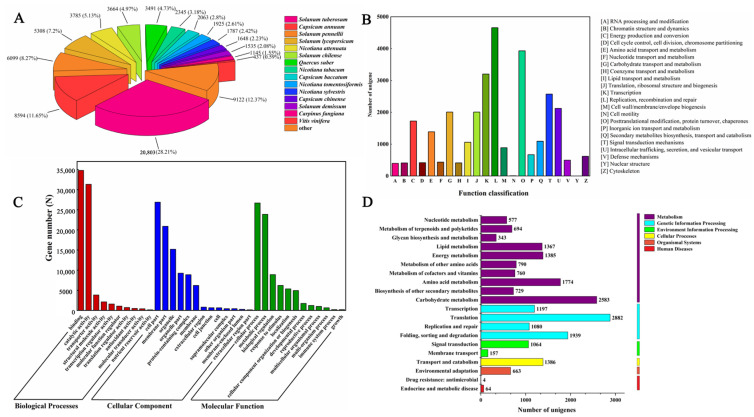
Functional annotation of unigenes in the transcriptome of *S. nigrum* during seed germination. (**A**) Nr annotation; (**B**) KOG annotation; (**C**) GO annotation; (**D**) KEGG annotation.

The assembled unigenes of *S. nigrum* achieved the highest annotation in the Nr databases, with 73,302 genes, which accounted for 50.71% of the total. Unigenes annotated in the other databases were KOG (59,257 genes; 40.99%), GO (59,121 genes; 40.90%), and KEGG (30,258 genes; 20.93%). With reference to species, the unigene sequences exhibited the maximum similarity to gene sequences from *S. tuberosum* (20,803), followed by *Capsicum annuum* (8594) and *S. pennellii* (9122; Figure 4A).

In the KOG database, the unigenes were divided into 22 groups, of which the 4 most annotated groups were as follows: [L] Replication, recombination, and repair (4657); [O] Posttranslational modification and protein turnover (3930); [K] Transcription (3202); and [T] Signal transduction mechanisms (2572; Figure 4B).

Annotated unigenes in GO were classified into three major categories: molecular function, cellular components, and biological processes. For molecular function, most genes were involved in binding and catalytic activity; for cellular components, most genes were involved in the cell part and membrane part; and for the biological processes, most genes were involved in cellular and metabolic processes (Figure 4C).

In KEGG, unigenes were annotated to five main divisions, which were metabolism, genetic information processing, environment information processing, cellular processes, and organismal systems. The leading metabolic pathways with the most unigenes enriched for the five divisions were carbohydrate metabolism (2583), translation (2882), signal transduction (1064), transport and catabolism (1386), and environmental adaptation (663), respectively (Figure 4D).

### 2.5. Analysis of Differentially Expressed Genes

**Figure 5:**
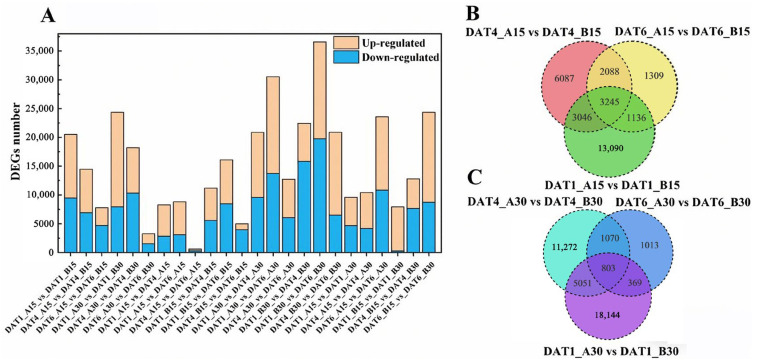
Differentially expressed genes (DEGs) between *S. nigrum* populations XJ1600 (**A**) and XJ1633 (**B**) during seed imbibition under 15 °C and 30 °C. (**A**) Number of DEGs in 24 comparison groups and number of up-regulated and down-regulated DEGs within groups. (**B**) Venn plots of DEGs from two population comparison groups at three imbibition timepoints at 15 °C. (**C**) Venn plots of DEGs from two population comparison groups at three imbibition timepoints at 30 °C.

Figure 5A illustrates the differentially expressed genes identified between two *S. nigrum* populations (XJ1600 and XJ1633) during seed imbibition under two temperature conditions (15 °C and 30 °C) at 1, 4, and 6 DAT. Unigenes with a *q*-value (adjusted *p*-value) < 0.05 and |log_2_(fold change)| ≥ 1 were defined as DEGs. The maximum DEG number (36,579) was obtained for the comparison group of the XJ1633 population at 1 DAT and 6 DAT under 30 °C (DAT1_B30 vs. DAT6_B30), which included 16,811 up-regulated genes and 19,768 down-regulated genes. The minimum DEG number obtained was 611, with 291 up-regulated genes and 320 down-regulated genes, for the comparison group of the XJ1600 population at 4 DAT and 6 DAT under 15 °C (DAT4_A15 vs. DAT6_A15). Furthermore, the Venn diagram illustrates the number of specific DEGs between the two populations at different imbibition conditions. Notably, when seeds were incubated at 15 °C, a total of 3245 common DEGs were consistently identified across all three comparison groups at different imbibition times (1, 4, and 6 DAT). However, for seeds incubated at 30 °C, a total of 803 common DEGs were identified (Figure 5B,C).

### 2.6. GO and KEGG Enrichment Analysis

**Figure 6:**
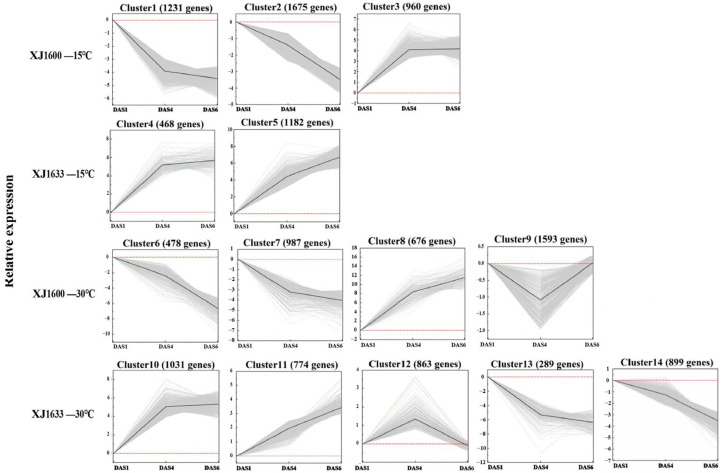
Cluster diagrams of DEGs based on pairwise comparisons. K-means clustering of DEGs for 4 groups was performed based on the Pearson correlation of gene expression profiles. The black line in each panel represents the mean pattern of DEG expression for each cluster. The DEGs in each cluster were examined with KEGG enrichment analysis based on the criterion of a correlated *p*-value ≤ 0.01.

Temporal profiling of differential gene expression was conducted in the XJ1600 and XJ1633 populations across three seed imbibition time points under two temperature conditions (15 °C and 30 °C). Based on the comparison datasets and the relative expression levels of DEGs, the expression patterns were classified into four groups (XJ1600—15 °C, XJ1600—30 °C, XJ1633—15 °C, XJ1633—30 °C) and 14 clusters (Figure 6). Along with the increasing imbibition time, gene expression generally increased in clusters 3, 4, 5, 8, 10, and 11 but decreased in clusters 1, 2, 6, 7, 13, and 14. For genes in clusters 9 and 12, their expression decreased or increased with increasing imbibition time but finally remained the same as at the beginning.

**Figure 7:**
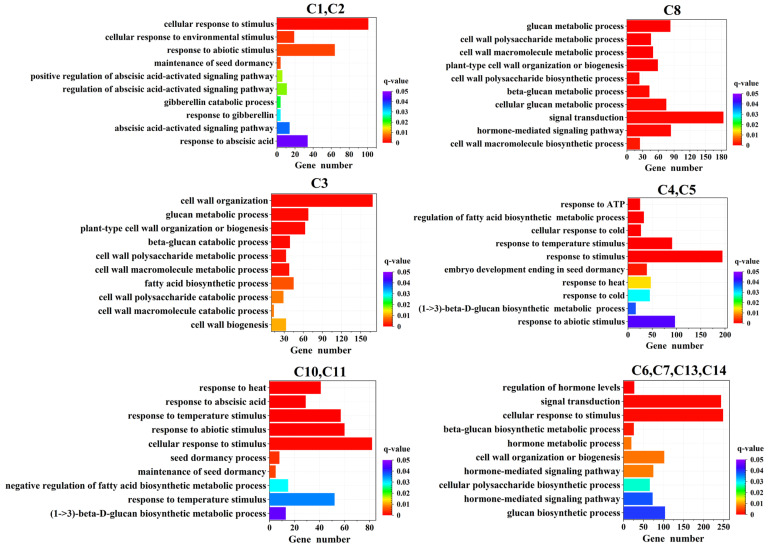
GO analysis of DEGs for different clusters in *Solanum nigrum* seeds (top 10 enrichment).

Functional enrichment analysis, including GO and KEGG, was performed to identify which DEGs were significantly enriched in GO terms and metabolic pathways at a Bonferroni-corrected *p*-value < 0.05 (sorted in ascending order of the *q*-value, *q* < 0.05). Based on the expression patterns of each group, the DEGs could be divided into six GO enrichment categories (Figure 7). Within the XJ1600—15 °C group, the down-regulated DEGs in C1 and C2 were primarily associated with a cellular response to a stimulus, a response to an abiotic stimulus, and a cellular response to an environmental stimulus. In contrast, the up-regulated DEGs in C3 were predominantly enriched in cell wall organization, the glucan metabolic process, and plant-type cell wall organization or biogenesis. In the XJ1633—15 °C group, the DEGs in C4 and C5 with up-regulated expression were mainly enriched in response to a stimulus, in response to a temperature stimulus, and in embryo development ending in seed dormancy. The up-regulated DEGs in C8 of the XJ1600—30 °C group were mainly enriched in signal transduction, a hormone-mediated signaling pathway, and the glucan metabolic process. For the XJ1633—30 °C group, the DEGs in C10 and C11 were mainly enriched in cellular response to a stimulus, in response to an abiotic stimulus, and in response to a temperature stimulus. The DEGs in C6, C7, C13, and C14 from the XJ1600—30 °C and XJ1633—30 °C groups were mainly enriched in cellular response to a stimulus and signal transduction.

**Figure 8:**
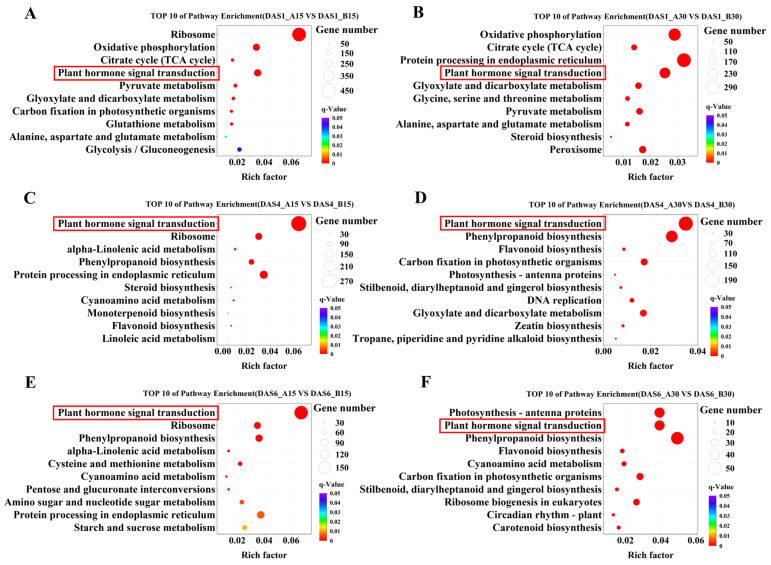
KEGG pathway enrichment analysis of DEGs in *S. nigrum* seeds (top 10 enrichment). (**A**) Top 10 of pathway enrichment (DAS1—A15 vs. DAS1—B15); (**B**) Top 10 of pathway enrichment (DAS1—A30 vs. DAS1—B30); (**C**) Top 10 of pathway enrichment (DAS4—A15 vs. DAS4—B15); (**D**) Top 10 of pathway enrichment (DAS4—A30 vs. DAS4—B30); (**E**) Top 10 of pathway enrichment (DAS6—A15 vs. DAS6—B15) (**F**) Top 10 of pathway enrichment (DAS6—A30 vs. DAS6—B30). The red box highlights the plant hormone signal transduction.

In KEGG analysis, DEGs of the two populations (XJ1600 and XJ1633) were significantly enriched in ribosome, oxidative phosphorylation, and plant hormone signal transduction when imbibed at 15 °C for 1 day (Figure 8A). At 4 DAT, the DEGs were significantly enriched in plant hormone signal transduction and ribosome and protein processing in the endoplasmic reticulum (Figure 8C). At 6 DAT, DEGs were significantly enriched in plant hormone signal transduction and ribosome and phenylpropanoid biosynthesis (Figure 8E).

At 30 °C for 1 day, DEGs of the two populations were significantly enriched in oxidative phosphorylation, protein processing in the endoplasmic reticulum, and plant hormone signal transduction. The DEGs were significantly enriched in plant hormone signal transduction, phenylpropanoid biosynthesis, and flavonoid biosynthesis at 4 DAT, and they were enriched in photosynthesis-antenna proteins, plant hormone signal transduction, and phenylpropanoid biosynthesis at DAT6. As the soaking time increases, plant hormone signal transduction becomes increasingly crucial for *S. nigrum* seed germination, suggesting that temperature-responsive germination is closely associated with the synthesis and signaling of plant hormones (Figure 8). Additionally, the activation of hormone pathways likely not only facilitates germination but also sets the stage for the subsequent coordinated action of other hormones like cytokinin, brassinosteroids, and salicylic acid to ensure optimal seedling establishment [8,21,22].

**Figure 9:**
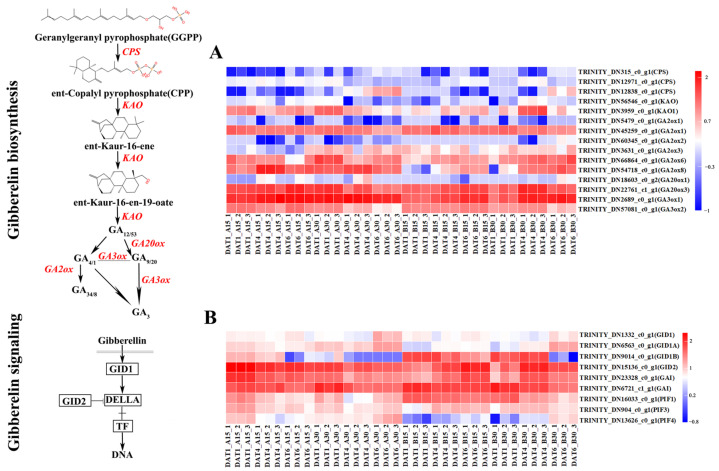
Regulatory model of DEGs in GA biosynthesis and signaling-related pathways during the seeding process. (**A**) Expression heat map of DEGs involved in GA biosynthesis. (**B**) Expression heat map of DEGs involved in GA signaling. Gradient colors represent a log10–fold change in gene expression under different treatments; red represents a high level of expression, whereas blue represents a low level of expression.

**Figure 10:**
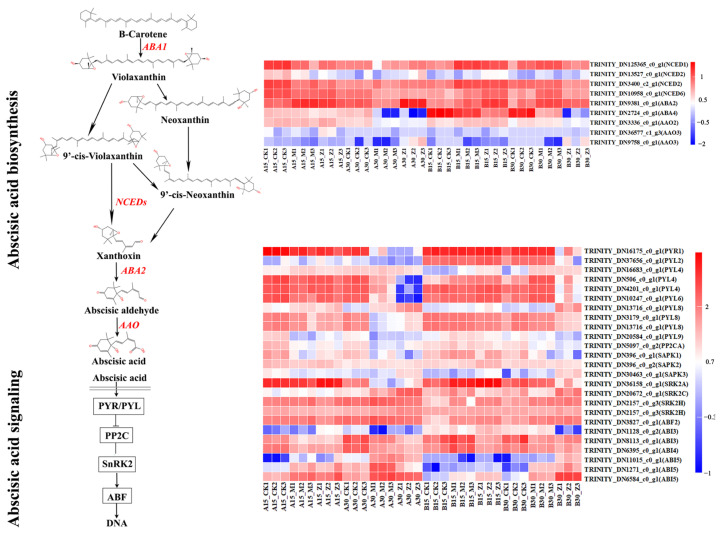
Regulatory model of DEGs in ABA biosynthesis and signaling-related pathways during the seeding process. (**Top**) Expression heat map of DEGs involved in ABA biosynthesis. (**Bottom**) Expression heat map of DEGs involved in ABA signaling. Gradient colors represent a log10–fold change in gene expression under different treatments; red represents a high level of expression, whereas blue represents a low level of expression.

Further analysis revealed that in the GA synthesis and signal transduction pathway, related GA enzymes, such as *GA2ox1*, which is involved in GA catabolism, were significantly down-regulated, while key enzyme genes *GA20ox3* and *GA3ox1* involved in GA biosynthesis were significantly up-regulated. The relative expression of *GID1B* gradually decreased with an increase in seed immersion time (Figure 9). In the ABA synthesis and signal transduction pathway, the relative expression of *PYL4* exhibited a gradual decline as the immersion time increased. In contrast, the expression of *ABI5* gradually decreased with an increase in immersion time at a low temperature and increased at a high temperature. Therefore, the GA and ABA synthesis and signal transduction pathways may be involved in the regulation of germination of *S. nigrum* seeds through temperature (Figure 10).

### 2.7. Candidate Gene Selection and Validation

**Figure 11:**
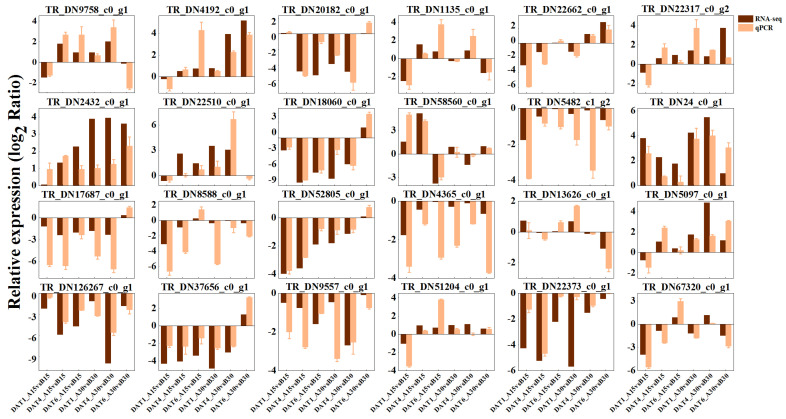
Validation of RNA–seq results of 24 DEGs with RT–qPCR.

We identified 24 DEGs in the *S. nigrum* transcriptome database for the validation of the transcriptome data. The above DEGs were mainly enriched in the cold response, abscisic acid response, seed germination, heat response, and gibberellin catabolic pathways in the GO database, as well as the phytohormone signaling pathway and MAPK signaling pathway in the pathway enrichment analysis. These genes are likely to play a critical role in the germination response to temperature in *S. nigrum* seeds. Using the *EF1α* as the reference gene, which was identified as exhibiting a stable expression in *S. nigrum* seeds, we quantified the relative expression levels of the target genes via RT–qPCR. The primer sequences for the genes are provided in Table 1. Six treatment groups were selected for RT–qPCR validation, and the expression trends are shown in Figure 11. It can be observed that there are slight discrepancies between the transcriptome sequencing results and the RT–qPCR validation fold changes. However, the overall trends remain consistent, demonstrating the high reliability and reproducibility of the transcriptome data.

### 2.8. Dynamics of Temperature-Responsive Gene Expression

Based on the transcriptome sequencing data, eight high-priority candidate genes involved in the temperature response were identified from DEGs (Table 2). These include GA biosynthetic genes: gibberellin 20-oxidase (*GA20ox1*) and gibberellin 3-beta-dioxygenase (*GA3ox1*); a GA catabolic gene: gibberellin 2-oxidase (*GA2ox1*); an ABA biosynthetic gene: 9-cis-epoxycarotenoid dioxygenase (*NCED9*); and an ABA catabolic gene: abscisic acid 8′-hydroxylase (*CYP707A2*), as well as key regulatory genes, such as *SPATULA* (*SPT*), phytochrome-interacting factors (*PIF1*), and abscisic acid-insensitive 5 (*ABI5*).

**Figure 12:**
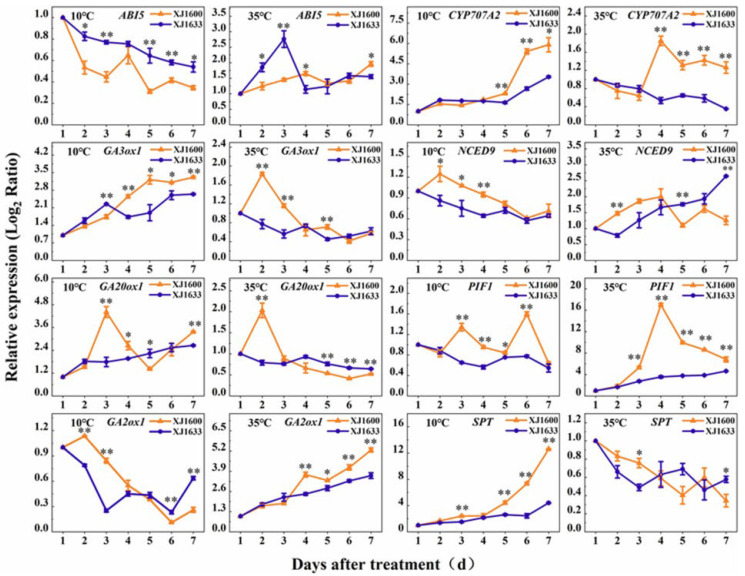
Expression levels of DEGs in response to temperature during the germination of *S. nigrum* seeds. * represents significant differences at the *p* ≤ 0.05 level. ** represents significant differences at the *p* ≤ 0.01 level.

To further elucidate the molecular mechanisms underlying the temperature-regulated germination of *S. nigrum* seeds (XJ1600 and XJ1633) and maximize the contrast for identifying temperature-responsive genes, we analyzed differential gene expression profiles from 1 to 7 days under two contrasting temperature regimes: a low temperature (10 °C) and a high temperature (35 °C) treatment (Figure 12).

In the ABA synthesis pathway, the gene involved in the biosynthesis, *NCED9*, was down-regulated in expression with increasing immersion time at a low temperature, and the difference was significant on DAT4. *CYP707A2*, a key gene encoding the ABA catabolic enzyme, exhibited significant up-regulation under the low-temperature treatment, and the expression was up-regulated by about 6–fold on DAT7. In contrast, at high temperatures, ABA catabolism-related genes displayed divergent expression patterns between the two *S. nigrum* populations. These findings suggest that high temperatures can induce seed dormancy by up-regulating ABA biosynthesis genes while down-regulating catabolic genes, whereas dormancy release may be associated with the up-regulation of catabolic genes over time. Conversely, low temperature likely promotes germination by suppressing the expression of negative regulators in ABA signaling.

In the GA biosynthesis pathway, the expression patterns of the two key biosynthetic genes (*GA3ox1* and *GA20ox1*) exhibited consistent trends. Notably, a significant divergence was observed between the expression of the catabolic gene (*GA2ox1*). Furthermore, the expression profiles under high-temperature conditions displayed an inverse trend compared to those under low-temperature conditions. In the GA signaling pathway, the expression of *SPT* was gradually up-regulated with seed immersion time at low temperatures. In contrast, under high-temperature conditions, *PIF1* expression was up-regulated by about 17–fold on DAT4, while *SPT* expression showed a down-regulation. We speculated that, within the GA signaling pathway, *SPT* acts as a positive regulator of low-temperature signaling, exhibiting up-regulated expression under low temperatures to promote germination. Conversely, at high temperatures, *PIF1* plays a dominant role and up-regulates expression to promote seed germination.

**Figure 13:**
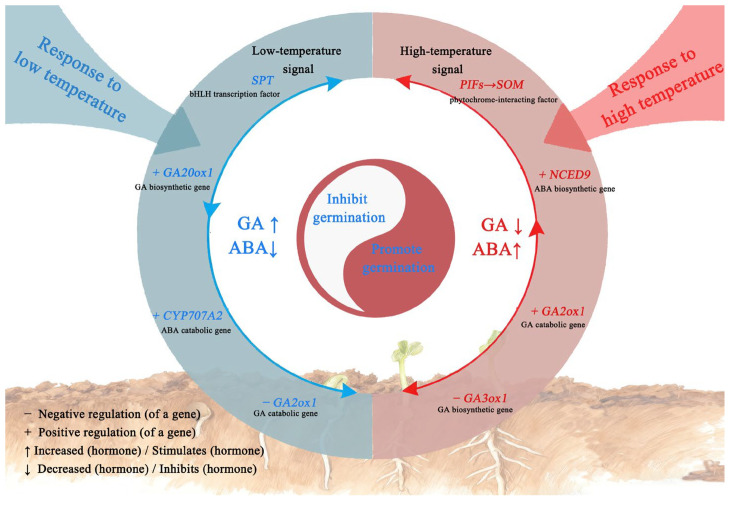
Regulatory mechanisms of germination-related genes in response to temperature in *S. nigrum* seeds.

Based on the previous studies and the regulatory relationships of the expression of temperature response-related genes, a preliminary regulatory pathway of *S. nigrum* seed germination in response to temperature signals was constructed [40]. Under low-temperature conditions, the low-temperature signal could be mainly sensed by *SPT*. This regulator up-regulated *GA20ox1* and *CYP707A2* but down-regulated *GA2ox1*, potentially enhancing seed germination by promoting bioactive GA levels and inhibiting ABA biosynthesis. Under high-temperature conditions, the *PIFs* appear to mainly sense the thermal signal and modulate seed germination in *S. nigrum* through a regulation involving *SOMNUS* (*SOM)*-mediated regulation. *SOM* is considered a key negative regulator of seed germination based on previous studies, suggesting that its role is primarily modulated by ABA and GA metabolism [41]. This involves up-regulating *NCED9* and *GA2ox1* expression while down-regulating *GA3ox1* expression, thereby altering the ABA/GA balance and contributing to the control of the germination of *S. nigrum* seeds (Figure 13).

## 3. Discussion

Seed germination is a highly intricate physiological process, wherein temperature serves a fundamental role in transitioning seeds from a state of relative dormancy to active physiological and metabolic growth [3]. The direct effect of temperature on seeds is reflected in the germination rate and speed. Studies have shown that an optimum temperature promotes peak seed germination performance [6]. In our study, the germination response of *S. nigrum* seeds collected from the same geographical location (XJ1600 and XJ1633) exhibited significant variation when exposed to different incubation temperatures. Our results demonstrated that 30 °C is more conducive to seed germination than 15 °C, which is consistent with the report by Ma et al. [20]. However, we further found that germination at 15 °C showed a significant reduction in germination rate and delayed germination speed compared to the optimal temperature. Based on our preliminary observations, we hypothesize that these phenomena might be associated with alterations in hormone metabolism and the regulation of gene expression. To verify this hypothesis, we conducted hormone content measurements and transcriptome sequencing analysis [42,43].

Temperature stands as a pivotal environmental factor governing seed germination, with a well-established correlation between thermal conditions and the dynamic regulation of endogenous phytohormones within seeds [13]. Hormone regulation of seed dormancy and germination may be a highly conserved mechanism among the seeds [44]. Critically, hormone levels should be viewed as a readout of the physiological status within the seed, rather than as direct actors in the regulatory network. This is exemplified by the antagonistic actions of ABA and GA: ABA promotes dormancy and inhibits germination, while GA counteracts ABA to promote germination [45]. Consequently, the decision to germinate is reflected in the ratio of these hormones, i.e., high ABA-to-GA ratios maintain dormancy, while low ratios promote germination [46]. In immature seeds, endogenous ABA maintained the process of seed embryo development rather than germination [47,48,49]. Pre-harvest germination before harvest was also associated with lower ABA contents in seeds. Notably, during the early days of imbibition, the ABA content decreased much more in non-dormant seeds than in dormant seeds [50]. The role of ABA in seed dormancy and germination is mainly regulated by ABA metabolism and signaling pathways, and environmental factors also affect seed dormancy and germination through ABA and GA interactions [51]. However, substantial evidence suggests that GA mainly functions to promote germination after dormancy has been alleviated, rather than directly inducing dormancy release [52]. In our experimental design, all samples for hormone measurement were harvested at the same time points across all experimental groups. Our study showed that GA plays a major role in the germination process of *S. nigrum* seeds, with germination initiating once the GA/ABA ratio exceeded 1, which is consistent with previous reports. Furthermore, we found that the GA content peaked at 30 °C after 4 days and was positively correlated with the germination rate of *S. nigrum* seeds. In contrast, the ABA content decreased to its lowest level within this temperature range and showed a negative correlation with germination. These findings suggest that the endogenous balance between GA and ABA plays a critical role in regulating seed dormancy and germination in *S. nigrum*. Specifically, GA acts as a positive regulator, with its synthesis and metabolic activity increasing markedly just prior to mass germination. Building on our findings regarding the central GA–ABA interplay, future work should employ more comprehensive techniques, like HPLC-MS, to elucidate the complete hormonal network.

Seeds perceive temperature variations through specific genes, enabling them to adapt to seasonal shifts and microenvironmental differences [53]. In previous studies, a temperature of 34 °C has been shown to inhibit seed germination in *Arabidopsis* by modulating phytohormone metabolism. Specifically, high-temperature conditions were found to promote ABA biosynthesis through the up-regulation of *NCED9* while simultaneously suppressing GA biosynthesis by down-regulating the expression of *GA20ox1* and *GA3ox1*, thereby creating a hormonal balance unfavorable for germination. Under supra-optimal temperatures, the expression of *NCED9* in *Arabidopsis* seeds was significantly up-regulated by 2.5– to 6–fold within three days [53]. In our study, although the initial screening of differentially expressed genes (DEGs) was based on |log_2_ (fold change)| and *q*-value thresholds, we subsequently focused on identifying key regulatory genes within hormonal pathways due to their disproportionate impact on the germination phenotype. With this focused approach, we found that under the high-temperature condition of 35 °C, the expression of *NCED9* in *S. nigrum* seeds was also significantly up-regulated, showing an approximately 3-fold increase in relative expression as the soaking duration extended. This up-regulation suggests that *NCED9* may play a pivotal role in high-temperature-induced ABA biosynthesis, which could subsequently lead to the inhibition of seed germination. Furthermore, the observed enhancement of ABA biosynthesis under elevated temperatures lends support to the hypothesis that *NCED9* serves as a critical regulator of ABA synthesis and germination suppression under thermal stress. In the XJ1600 population, the expression of *CYP707A2* was transiently down-regulated within 2 d of the high-temperature treatment, and the relative expression was up-regulated by about 2–fold at 3–7 d. The expression was down-regulated by about 3–fold in the XJ1633 population. However, our results differ slightly from those of Toh et al. [54], who reported that in *Arabidopsis* seeds, *CYP707A2* transcripts were translated within 6 h prior to imbibition and that the high-temperature treatment transiently up-regulated their expression. This discrepancy could be attributed to differences in seed dormancy and germination strategies between species. Furthermore, we now argue that the value of our model lies not in describing inhibition at 35 °C but in its consistency and predictive power across a temperature gradient. The fact that the same core set of genes shows a coordinated, temperature-dependent expression shift (from 10 °C to 35 °C) provides a plausible transcriptional basis for the observed physiological response curve. As demonstrated in Figure 12, the expression profiles of both *GA20ox1* and *GA3ox1* exhibit a congruent, temperature-dependent trajectory.

A previous study by Yamauchi et al. suggested that *GA20ox1*, *GA3ox1*, and *GA2ox1* play important roles in mediating the low-temperature response [31]. During seed imbibition, the low-temperature treatment up-regulates the transcript levels of *GA20ox1* and *GA3ox1* while suppressing *GA2ox1* expression, thereby activating the gibberellin biosynthesis pathway [31,55]. In our study, low temperatures up-regulated the expression of *GA20ox1* and *GA3ox1* in both *S. nigrum* populations. Their transcript levels increased by approximately 5–fold over the treatment period, suggesting that this up-regulation could enhance endogenous GA content in seeds and promote germination through stimulated GA biosynthesis. In contrast, *GA2ox1* expression was down-regulated under low temperatures, with the XJ1600 population exhibiting the lowest relative expression (approximately 10–fold reduction) 6 days after treatment. Under the high-temperature treatment, *GA20ox1* and *GA3ox1* maintained low biological activity. Their expression levels were briefly up-regulated within 2 d in the XJ1600 population but showed significant down-regulation after 2 d of immersion. In contrast, the relative expression of *GA2ox1* was up-regulated and gradually increased with an increase in immersion time. This shift in gene expression suggests a potential mechanism for the fine-tuning of GA homeostasis under different temperature conditions. However, in the XJ1600 population at 30 °C, this regulatory activity may not have been sufficient to overcome the overall promotion of germination, which appears to have been driven by a concurrent peak in bioactive GA content, as shown in Figure 2. A more pronounced shift in gene expression under higher stress temperatures corresponds with the significant suppression of GA and inhibition of germination. This phenomenon can be linked to ABA, which plays a crucial role in inhibiting GA biosynthesis during seed germination [45,46]. Since high temperatures are known to amplify the inhibitory effect of ABA in seeds [56], the enhanced ABA activity likely contributes to the observed down-regulation of GA20ox1 and GA3ox1, consequently inhibiting GA synthesis.

Transcription factors, such as *SPT* and *PIFs*, may indirectly regulate seed germination through *SOM* and *ABI5* in response to environmental temperature signals [57,58]. Previous studies have shown that *SPT* is also involved in the response of seed germination to low temperatures, and one of the key aims of seed cold signaling is to promote GA biosynthesis through the transcriptional regulation of *GA3ox* [57]. Under low-temperature conditions, *SPT* expression was significantly up-regulated with prolonged duration in both *S. nigrum* populations, suggesting a positive regulatory role in seed germination during low-temperature stratification. Conversely, under high-temperature conditions, the relative expression of *SPT* was significantly down-regulated in both populations. This study aligns with prior research suggesting that a low-temperature treatment up-regulates *GA3ox1* transcription by promoting the expression of *SPT*, thereby potentially stimulating GA biosynthesis to facilitate seed germination.

This study provides preliminary insights into the molecular mechanism by which temperature signals regulate seed germination in *S. nigrum* through a core transcriptional network that modulates the ABA/GA balance. These findings offer new perspectives for weed management in agricultural ecosystems. In crop systems such as cotton and spring maize, sowing during periods when soil temperatures remain below 15 °C could leverage the weak germination vigor of *S. nigrum* at low temperatures, thereby providing crops with an early competitive advantage. Furthermore, the temperature-responsive candidate genes identified in this study represent potential targets for developing precision herbicides capable of disrupting weed germination without adversely affecting crops. Subsequent research should focus on generating integrated transcriptome and hormone profiles under fully inhibitory temperatures, utilizing developmental stage-based sampling to precisely capture physiological transitions. Functional analysis of the prioritized candidate genes will be essential to definitively establish their roles in this regulatory network.

## 4. Materials and Methods

### 4.1. Experimental Design

To elucidate the mechanism by which temperature regulates seed germination in *S. nigrum* via phytohormone pathways, a two-stage targeted strategy was employed in this study.

First, the phenotypic boundaries of germination were defined through an initial assay across a broad temperature gradient (10–35 °C). The results confirmed that 15 °C and 30 °C were key temperatures supporting successful germination, whereas 10 °C and 35 °C exhibited significant germination inhibition.

Based on this phenotypic profile, 15 °C (sub-optimal) and 30 °C (optimal) were selected for transcriptomic analysis. This comparison leveraged their “same-success-but-different-efficiency” characteristic to identify potential candidate genes governing germination vigor and efficiency, with a focused investigation of the ABA and GA pathways.

Subsequently, candidate genes screened from the ABA and GA pathways were subjected to qRT–PCR analysis under 10 °C (low-temperature stress) and 35 °C (high-temperature stress). This step aimed to investigate the correlation between their expression changes and the observed germination inhibition, thereby evaluating the potential role of these genes in the broader context of temperature stress response.

### 4.2. Plant Growth Conditions

Seed samples of two *S. nigrum* populations (XJ1600 and XJ1633) were collected from a cotton field in Xinjiang, China (44°51′56″ N, 82°20′33″ E) in 2018. In previous germination studies, these two populations showed distinct germination behavior under different temperature regimes [20]. All seeds were sterilized with a 2% sodium hypochlorite solution for 10 min and thoroughly rinsed with sterile distilled water.

### 4.3. Seed Germination

The sterilized seeds from two *S. nigrum* populations (XJ1600 and XJ1633) were then transferred to growth incubators (Ningbo Jiangnan Instrument Factory, Ningbo, China) set to temperatures of 10 °C to 35 °C. The incubation conditions were maintained at a 12 h light/12 h darkness photoperiod with 50% relative humidity. Each population was analyzed using 3 replicates of 40 seeds per Petri dish. The number of germinated seeds was recorded daily from 0 to 10 days. Germination was defined as the radicle emerging ≥1 mm from the seed coat. The germination rate was calculated using the following equations: Germination rate (%) = G_a_/G_n_ × 100, where G_a_ is the number of all germinated seeds at the end of germination and G_n_ is the total number of seeds tested; Germination index = ∑(N_t_/D_t_), where N_t_ is the number of seeds germinated on day t, and D_t_ represents the corresponding day of germination [20]. Based on the seed germination rates and hormone concentration data, the most suitable treatment group was selected for subsequent experiments.

### 4.4. Determination of GA and ABA Content

Seed samples were collected from two *S. nigrum* populations (XJ1600 and XJ1633) for GA and ABA content analysis. These samples were obtained after seed imbibition for 0–10 d under two distinct temperature conditions (15 °C and 30 °C). *S. nigrum* seeds (0.1 g fresh weight) were ground in phosphoric acid buffer in an ice bath and centrifuged at 8500 rpm and 4 °C for 10 min, and the supernatant was collected for reserve. ABA and GA content were determined by an enzyme-linked immunosorbent assay (ELISA) according to the instructions of the kit (Mlbio, Shanghai, China). All experiments were carried out with a minimum of 3 replicates to ensure consistency and accuracy.

### 4.5. Transcriptome Sequencing

A total of 36 seed samples were collected from two *S. nigrum* populations (XJ1600 and XJ1633) for deep sequencing analysis. These samples were obtained after seed imbibition for 1, 4, and 6 d under two distinct temperature conditions (15 °C and 30 °C) and included ungerminated and germinated seeds. Total RNA was extracted from *S. nigrum* seeds using a plant RNA purification reagent for plant tissue according to the manufacturer’s instructions (Invitrogen, Carlsbad, CA, USA), and genomic DNA was removed using DNase I (TaKara, Tokyo, Japan). Then, the RNA quality was determined using a 2100 Bioanalyser (Agilent Technologies, CA, USA) and quantified using the ND—2000 (NanoDrop Technologies, Foster City, CA, USA). The sequencing library was prepared according to the TruSeq^TM^ RNA sample preparation Kit from Illumina (San Diego, CA, USA) using 1 μg of total RNA. Double-stranded cDNA was synthesized using a SuperScript double-stranded cDNA synthesis kit (Invitrogen, Carlsbad, CA, USA) with random hexamer primers (Illumina, San Diego, CA, USA). After being quantified by TBS380, a paired-end RNA—seq library was sequenced with the Illumina HiSeq XTen/NovaSeq 6000 sequencer (Illumina, San Diego, CA, USA) (2 × 150 bp read length).

### 4.6. GO Enrichment Analysis

The assembly of clean reads was performed in Trinity. Unigenes were annotated in four databases: NCBI non-redundant protein sequence database (Nr), KEGG orthology database, gene ontology (GO), and clusters of orthologous groups of proteins (KOG). To identify differentially expressed genes (DEGs) between two different temperature regimes, gene expression levels were calculated as fragments per kilobase of exon model per million mapped reads (FPKM). The DEseq2 algorithm was adopted to improve the differences in genetic testing and screening. The parameters |log_2_ (fold change)| ≥ 1 and *q*-value (adjusted *p*-value) < 0.05 were used to identify DEGs [58,59]. Functional enrichment analysis, including GO and KEGG, was performed to identify which DEGs were significantly enriched in GO terms and metabolic pathways at a Bonferroni-corrected *p*-value < 0.05. GO functional enrichment and KEGG pathway analysis were carried out using Goatools and KOBAS [60].

From the resulting DEGs, a subset of eight candidate genes was selected for further experimental validation. This selection was based on a combination of their high statistical significance (particularly a high fold change), their central roles in the significantly enriched GO terms and KEGG pathways related to phytohormone signaling and seed germination (as outlined in the results), and their established biological importance in these processes based on the existing literature.

### 4.7. RT–qPCR Analysis

Seeds of *S*. *nigrum* populations XJ1600 and XJ1633 were subjected to temperature treatments of 10 °C and 35 °C for 1–7 days. Samples were collected at seven time points in triplicate, flash-frozen in liquid nitrogen, and stored at −80 °C for qPCR Analysis.

The RNA–seq data were verified by RT–qPCR analysis. Total RNA was extracted from independent samples with an RNAprep Pure Plant Kit (Tiangen Biotech, Beijing, China). Total RNA (1 μg) was reverse-transcribed using FastKing gDNA Dispelling RT SuperMix (Tiangen Biotech, Beijing, China). All RT–qPCR primers (Table 1) were designed using SnapGene^®^ 4.3.6 software. RT–qPCR was conducted with Talent RT–qPCR PreMix (SYBR Green) (Tiangen Biotech, Beijing, China) in a 20 μL reaction volume containing 10 μL of RT–qPCR PreMix, 0.6 μL of forward and reverse primer, 1 μL of cDNA, and 7.8 μL of ddH_2_O. The RT–qPCR amplification was performed under the following conditions: initial denaturation at 95 °C for 3 min; 40 cycles of denaturation at 95 °C for 5 s, annealing at 60 °C for 10 s, and extension at 72 °C for 32 s. Quantitative PCR was performed using a Bio-Rad CFX96 Touch™ real-time PCR detection system (Bio-Rad Laboratories, Inc., Hercules, CA, USA). *EF1α* was regarded as a reference gene to normalize the expression level of the target genes. The raw data were analyzed using ABI 7500 software (version 2.3, Applied Biosystems, Foster City, CA, USA), and the expression level was obtained using the 2^−ΔΔCt^ method. The experiments were performed in three individual biological replicates [23].

### 4.8. Statistical Analysis

The data were analyzed using SPSS software (v.19.0; IBM, Armonk, NY, USA). One-way analysis of variance (ANOVA) was carried out, followed by Duncan’s multiple range tests (*p* < 0.05).

## 5. Conclusions

This study identified 73,314 annotated unigenes from *S. nigrum* transcriptomic data. As a primary finding, our data point to the likely involvement of the GA/ABA signaling pathways in the temperature-responsive regulation of seed germination. Furthermore, transcriptome sequencing, combined with RT–qPCR validation, confirmed eight high-priority candidate genes mediating this response. Under low-temperature conditions, *SPT* likely acts as the primary cold sensor, positively regulating *GA20ox1* and *CYP707A2* while negatively regulating *GA2ox1*, thereby potentially activating bioactive GA accumulation and suppressing ABA biosynthesis to enhance germination. Under high-temperature conditions, *PIFs* appear to be the main heat sensors, up-regulating *NCED9* and *GA2ox1* and down-regulating *GA3ox1* via SOM-mediated regulation, collectively modulating germination through the ABA/GA balance. Our findings help to characterize a temperature-responsive pathway in which SPT and PIFs appear to relay thermal signals to key hormone metabolic genes, a process correlated with dynamic changes in the GA/ABA ratio during germination. This work offers initial insights into the transcriptional network regulating *S. nigrum* germination under temperature shifts, laying the groundwork for future research. The genes we identified represent core components of this response, and uncovering the upstream regulators that integrate these signals is a clear and important next step stemming from this screening work.

## Figures and Tables

**Table 1:** Primers of 24 DEGs from *S. nigrum* seeds.

Genes	Forward Primer	Reverse Primer
TR_DN9758_c0_g1	ATCTTCGGCTAAGCAGGTTGTGG	GGCAGTCTGGTGGTGATGGAATG
TR_DN4192_c0_g1	GCTCGCTTACGCTTCCTCCAC	CCGTTGTTCTGTTGCTGCTGTTG
TR_DN20182_c0_g1	TGCAGGAAGAAGGAGGAGCT	TGACTTGATCTTGCACCTTGGT
TR_DN1135_c0_g1	GGCACTTCACAGACCAGGAACAC	CTTCTCCACCGTGGTTGTCATCC
TR_DN22662_c0_g1	CCATTCTTCGCACCGCCTACG	GTGAGATTCCTCCATTCCGCTGAG
TR_DN22317_c0_g2	TAGCCGCCTAGCCCACAACTAC	AGAAGGTCTACAGCCAGCGATCC
TR_DN2432_c0_g1	ACGGAATGGAAATGCTTCGAGACG	TCCTCACCACATTCTCTGCTGTTC
TR_DN22510_c0_g1	GGCAGAGTTAGCGTCCTTCGTG	AAGCCATCAGTAGCAAGCCTCAAC
TR_DN18060_c0_g1	GCCACAAGCAGTTGTCGAATTAGC	CGCCACTTGCTCTAGTACTCGTAG
TR_DN58560_c0_g1	GGATCAGCCGCAGCTCTTTCG	GACACCTGTTGCCTCTCCATTTCC
TR_DN5482_c1_g2	GGGCTTGAGAAAGAGGTTGAGTGG	TTGCTGCCGCTGCTCCATAATC
TR_DN24_c0_g1	GCGGAGGAGGAGGAGGAACAG	GCTTCTCAATCTCTGCTGCCTCAC
TR_DN17687_c0_g1	AGGGAAGCCGAGTCTATGTCAAGG	TGTCCATAAGCCGAGAGACCTCAG
TR_DN8588_c0_g1	AGCACAGCAAGCAAGGGAAAGG	GGACAGCCTCTCTTCAAGTTCAGC
TR_DN52805_c0_g1	TGGGTTTGGATTTGGTGGTTGGG	GGCGGAGGTGGTATGGGAAATTG
TR_DN4365_c0_g1	ATCCATTAGGCGTCACACCAACTG	CAGGATACCCAGCATCAGCCAAAC
TR_DN13626_c0_g1	CTACTGCGACGAATAGCCAGAGC	ACGGCTCCTTCGGGTAGTTCC
TR_DN5097_c0_g1	ATCGTGTCCAACTGCGGTGATTC	GTTCATCGGGTCTGTCAGGCTTG
TR_DN126267_c0_g1	AAACCCGTCTCCCTTCTCTCCATC	TTGCTGTTCCTGTTCCTGTGAGTG
TR_DN37656_c0_g1	TGACAGGTGACGGTGGAGTTGG	TCAGCCTATGTTCTCCGCCTACG
TR_DN9557_c0_g1	CATGCCATCTGATCCGACTCATCC	GATCCTGCTCCTCCTCCTCTTCTC
TR_DN51204_c0_g1	GCTTCTTGTGGATCGGCGATTTTC	TCCTTGTGACAGAACCTCCTCAGC
TR_DN22373_c0_g1	ACACAGAGAAGTGGATGGGTTAAGT	GCTTCTAATAATGGCAGCTTGAGCA
TR_DN67320_c0_g1	GGTGGTGATGAAGATGGAGTGGTG	CGCCGTTCTCCACCGTAACATAG

**Table 2:** Primers for the response of 8 genes to temperature.

Genes	Forward Primer	Reverse Primer
*SPT*	CATGCCATCTGATCCGACTCATCC	GATCCTGCTCCTCCTCCTCTTCTC
*PIF1*	CTACTGCGACGAATAGCCAGAGC	ACGGCTCCTTCGGGTAGTTCC
*ABI5*	GCTCGCTTACGCTTCCTCCAC	CCGTTGTTCTGTTGCTGCTGTTG
*NCED9*	ACATAAGCTGAACCGGACAGTT	ATTTAACGGCGTGAATCATAC
*CYP707A2*	CGGCGGTTATGACATTCCTAAGGG	TCTGAACTCCAACGGGTCCTTCC
*GA2ox1*	ACCTCATTGTTAAGGCTTGCGAAGA	TTGTCGATACGAGGATGTGTTCGAC
*GA20ox1*	TGCATCCCATATGAAACGTGAGTAC	GTTGACTACAAGAAAGAAACCGTGA
*GA3ox1*	CCACCAACTCCATCAGCTCAGC	ATTTTGAGTCGGAAAGAGAGATGAG

## Data Availability

Data are available from the corresponding author upon reasonable request.
